# Anthropogenic Sources Dominate Foliar Chromium Dust Deposition in a Mining-Based Urban Region of South Africa

**DOI:** 10.3390/ijerph19042072

**Published:** 2022-02-12

**Authors:** Sutapa Adhikari, Anine Jordaan, Johan Paul Beukes, Stefan John Siebert

**Affiliations:** 1 Unit for Environmental Sciences and Management, North-West University, Potchefstroom 2520, South Africa; Stefan.Siebert@nwu.ac.za; 2 Laboratory for Electron Microscopy, Chemical Resource Beneficiation (CRB), North-West University, Potchefstroom 2520, South Africa; Anine.Jordaan@nwu.ac.za; 3 Chemical Resource Beneficiation (CRB), North-West University, Potchefstroom 2520, South Africa; Beukes@steinert.com.au

**Keywords:** air pollution, chromite, ferrochrome smelter, particulate matter, plant morphology, road dust, wind movement

## Abstract

Dust pollution can be severe in urban centers near mines and smelters. Identification of dust sources and assessing dust capturing plant morphological traits may help address the problem. A chromium (Cr) mining and ferrochrome smelting region in Sekhukhuneland, South Africa, was investigated to identify the sources of Cr in soil and plant leaf surfaces and to evaluate the association between Cr sources and plant morphology. Combinations of bi- and multivariate statistical analysis techniques were applied. Non-significant relation between Cr quantities in surface soil and on leaf surfaces suggested negligible Cr dust contribution from soil to leaves. Association among Cr, Fe, Mg, Al, and Si levels on leaf surfaces confirmed their shared origin, possibly from chromite containing dust dispersed by mines, smelters, roads, and tailings. Both plant morphology and Cr sources (number and proximity to mines and roads) conjointly determined Cr dust deposition on leaf surfaces. Air mass movement patterns further identified local polluters, i.e., mines, ferrochrome smelters, and roads, as dominant dust sources in the region. Common plant species showed Cr dust adhesion favouring traits (plant tallness, larger leaf area, dense epicuticular wax structures, and larger stomata) and projected dust mitigation prospects for Sekhukhuneland.

## 1. Introduction

Particulate matter (PM) containing hazardous elements are generated by various natural processes, but multifaceted anthropogenic activities accelerate the influx and widespread distribution of such particles in urban environments [1,2,3]. The spatial–temporal distribution range of PM could be extensive due to high-speed dispersion by wind depending on the aerodynamic diameters of the particles [4,5,6]. Mining and smelting operations emit large volumes of PM and are considered among the top ten pollution challenges worldwide [3,5,7]. Various stages of mining operations such as excavation, extraction, transportation of ore and waste materials, and dispersion of dust particles from open mine pits and tailings, may cause severe air pollution at nearby, or even distant localities [3,4,6,8,9]. Furthermore, environmental degradation and hazardous dust generation possibilities remain high for abandoned mines and tailing dams [10,11]. Consequently, PM pollution has been a global health threat to urban population, specifically in mining localities [3,9,12,13,14]. 

Vegetation cover not only resists wind movement and restricts further distribution of dust [15], but may also reduce emission of hazardous dust particles from contaminated sites such as tailings [6]. Furthermore, besides absorbing contaminants from soil, plants remove significant quantities of pollutants from the atmosphere primarily as a result of dry/wet deposition onto foliar surfaces [2,15,16,17,18]. Foliar dust capture capacity of plant species depends on morphological traits, specifically leaf macro- and micromorphological features (i.e., leaf area, epicuticular wax, and density and size of stomata and trichomes) [15,19,20,21,22]. However, proximity and type of pollution sources affect the quantitative and qualitative aspects of dust [3,5,6,23]. It can, therefore, be deduced that plant leaf surface–dust interactions are dominated by two main factors, plant morphology and pollution sources (e.g., proximity, and types and number of emitters).

Outdoor and indoor air pollution and PM linked health hazards have been a concern for active and abandoned mining and waste sites in South Africa [8,24,25]. For the chromium (Cr) and platinum (Pt) mining and ferrochrome smelting region of Sekhukhuneland in South Africa, emission from various pollution sources has led to differential spatial–temporal distribution of PM in the regional air mass [26]. For this region, aerial concentrations of Cr particles exceeded annual limits set for tri- (Cr(III)) and hexavalent (Cr(VI)) forms by New Zealand [23]. Furthermore, in this urbanized region [27], Cr dust deposition on plant leaf surfaces and leaf morphological traits that enhance foliar dust deposition has been reported [28]. Still, the interaction patterns between plant morphology and pollution sources and possible sources of Cr in the soil and plant leaves have never been explored for Sekhukhuneland. However, it is important to evaluate if anthropogenic activities dominate Cr dust deposition in soil–plant systems as dust generated by Cr mines and ferrochrome smelters may contain Cr(VI), which could be highly toxic to living organisms [29,30]. Accordingly, the two objectives of the present study were (1) to identify the possible sources of Cr in the soil and on plant leaf surfaces, and (2) to investigate the association between selected plant morphological traits (i.e., plant height, leaf area, epicuticular wax, and size and density of stomata and trichomes) and Cr sources (i.e., proximity to and the number of polluters) to assess leaf surface–Cr dust particle interactions. Air mass movement patterns were considered to further elucidate dust distribution in the region.

## 2. Materials and Methods

### 2.1. Study Area and Sampling 

**Figure 1:**
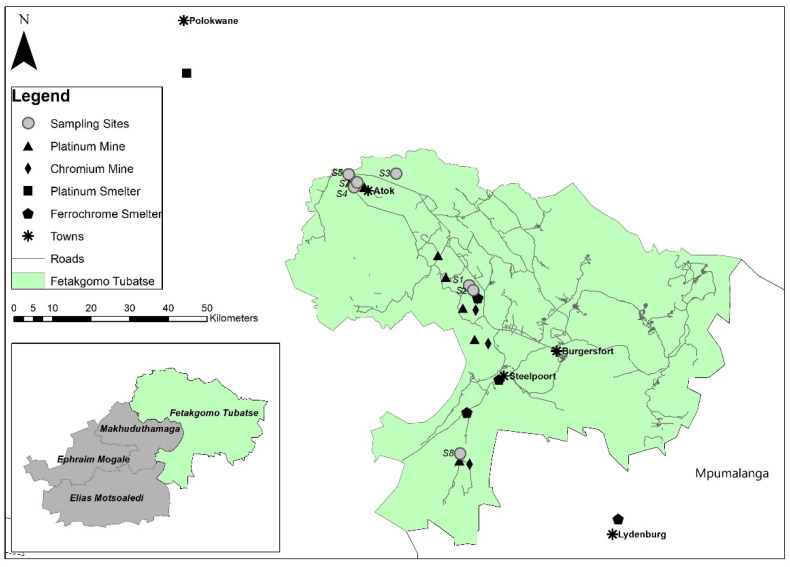
Location of the sampling sites (S1–S5, S7, S8), Cr and Pt mines, ferrochrome and Pt smelters, and roads in the Fetakgomo-Tubatse municipality in Sekhukhuneland, South Africa.

The study was conducted in the urbanized industrial region of Fetakgomo-Tubatse municipality of the Sekhukhune District in Limpopo Province, South Africa (Figure 1). Apart from several functional Cr and Pt mines, the district has three active ferrochrome smelters. Pt ore is also a Cr ore and Cr is extracted from Pt tailings [31], hence the Pt industry further contributes to Cr pollution. There are two other Pt and Cr smelters located in the nearby towns, Polokwane and Lydenburg. Mining and industrial growth have led to the expansion of transportation routes that intersect urban settlements. The study area also features the Rustenburg Layered Suite (RLS) and ultramafic soils naturally rich in Cr. The semi-arid climatic conditions of Sekhukhuneland could enhance dry dust suspension and retention in the lower atmosphere. The hilly terrain influences wind movement and dispersion of PM further in the region [26].

Leaves of 12 common home garden-grown and roadside plants were sampled during summer (November 2018). A total of seven urban localities in the vicinity of Cr and Pt mines and ferrochrome smelters in Sekhukhuneland were selected randomly (Table 1). Leaves were collected from five mature plants to form a composite per species. For each site, four sets of surface soil (0.1 m) collected from the base of sampled plants were mixed to form a composite. After air-drying thoroughly, soil samples were passed through a 2 mm sieve.

### 2.2. Leaf Analysis

**Figure 2:**
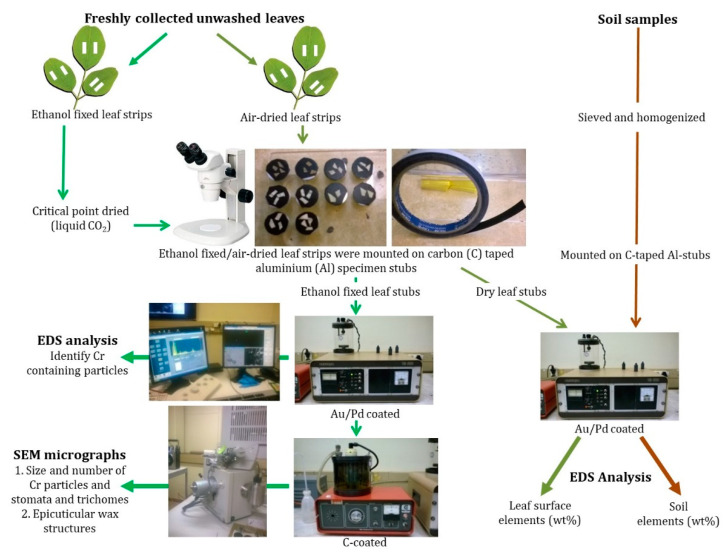
Outline of leaf and soil sample preparation and SEM-EDS analysis followed in this study. SEM-EDS, Scanning electron microscopy-Energy dispersive X-ray spectroscopy.

Sampled leaves were prepared and analyzed with energy dispersive X-ray spectroscopy (EDS) and scanning electron microscopy (SEM) (Figure 2) [28]. An Oxford X-map 20 EDS (Oxford Instruments, Abingdon, UK) detector and INCA (Integrated Calibration and Application Tool, ETAS, Stuttgart, Germany) software integrated into an FEI Quanta 250 FEG SEM (FEI, Bend, OR, USA) were used to quantify (weight percentage, wt%) Cr and other abundant elements (i.e., Al, Ca, Fe, K, Mg, and Si) on adaxial and abaxial leaf surfaces (0.01 wt% was considered the lowest element detection limit). On each leaf surface, dust particles were selected randomly to record the elements by using EDS analysis to identify Cr containing particles. SEM was used to micrograph identified Cr particles and such particles were counted and measured. SEM micrographs of leaf surfaces were further used to study foliar micromorphology and measure (lengths) and count stomata and trichomes and identify epicuticular wax structures. All counts and measurements were conducted using ImageJ software (Java-based image processing program) on five randomly chosen areas of 200 mm^2^ per leaf surface.

### 2.3. Soil Analysis

Sieved surface soils were prepared by homogenizing samples using a porcelain pestle and mortar. Ground sample per site was mounted on carbon (C) taped aluminium (Al) specimen stubs, coated with gold/palladium (Au/Pd) and analyzed at 15 kV and 10 mm working distance with the SEM-EDS system introduced in Section 2.2 (Figure 2). The most abundant elements, i.e., Al, Ca, Cr, Fe, Mg, and Si, in surface soils were quantified.

### 2.4. Plant Morphology

Plant heights were recorded for five individuals per species and means were calculated. Leaf area was calculated for five mature leaves per species using the formula, LA = GN × GA, where LA represents the leaf area, GN is the number of grids, and GA is the area of a single grid. Leaf area means were calculated thereafter.

### 2.5. Data Analysis

Pearson correlations, principal component analysis (PCA), and cluster analysis were performed to identify Cr sources in soil and plant samples [32,33]. Pearson correlation analysis was applied to determine the relationships between the abundant elements detected (1) in soil and (2) on both leaf surfaces. For both soil and plant leaves, PCA was conducted to evaluate the association among the elements, while cluster analysis was applied to identify homogenous groups of elements. Correlation matrix and Varimax rotation were used for PCA. Ward linkage method and Euclidean distance were applied to create dendrograms. Pollution sources and plant morphological traits were considered as the two variables to evaluate leaf surface–Cr dust interactions. As most of the sampling locations in the study region were in proximity to multiple Cr sources, both distances and the number of Cr and Pt mines (≤20 km) and roads (≤110 m) were accounted for and denoted as mine and road frequency, respectively. Smelters were excluded because only three sampling sites (S1, S2, and S8) were located in close vicinity (<13 km) of the three ferrochrome smelters in the study region (Table 1). Mine and road frequency indices were constructed based on five predetermined distance zones for (1) mines (km): 0–3, 3–6, 6–9, 9–15, 15–20, and (2) roads (m): 0–5, 5–15, 15–25, 45–15, and 45–105. Accordingly, values from 5 to 1 were assigned for the nearest to the most distant zone. Each sampling location was then scored by summing values obtained for the total number of mines and roads within each distance zone. Mine and road frequency was used as pollution variables. Selected plant morphological traits (i.e., plant height, leaf area, epicuticular wax, and size and density of stomata and trichome) were considered as plant variables. From SEM micrographs, the density of the epicuticular wax crystalloid structures was categorized in values ranging from 1 to 4. A value of 4 represented very dense; 3, dense; 2, moderate; 1, low; and 0, undetected wax structures. Pearson correlations were performed to determine relations between the plant and pollution variables with regards to Cr amount (Cr wt%) in particles and size of Cr particles (CrD). Factor analysis (FA) was conducted as a dimension reduction process to extract the most correlated variables (i.e., plant and pollution) with Cr wt% and CrD. FA identified the latent variables and extracted new factors based on the shared variance [34]. Varimax rotation, Kaiser normalization, and Principal axis factoring method were used for FA. Data were tested for homogeneity and standardized using z-score before analyses. FA, PCA, and Pearson correlation coefficient analyses were performed using STATISTICA version 7.0 (TIBCO Software, California, USA) and cluster analyses were conducted in IBM SPSS for Windows, version 27.0 (IBM Corporation, New York, USA).

### 2.6. Air Mass Movement Patterns

To assess the influence of wind on dust distribution, air mass movements were determined for the study area by calculating back trajectories. These back trajectories represent the movements of air parcels over space and time towards the sampling sites. The seven sampling sites were grouped as Area 1 (incorporated sampling sites S3, S4, S5 and S7), Area 2 (S1 and S2), and Area 3 (S8) based on their proximity to one another. 96-h back trajectories arriving at a height of 100 m above ground level, for every hour from 1 April 2018 to 30 November 2018 (sampling month), for each of the three areas were modelled. The calculation period considered time since the last significant rain (>15 mm on 23 March 2018), which could have washed away dust from leaf surfaces to start a new deposition cycle [35] until the end of the sampling month (November 2018). The Hybrid Single-Particle Lagrangian Integrated Trajectory Model (HYSPLIT, version 4.8) [36] was used for this purpose. Meteorological data of the Global Data Assimilation System (GDAS) archive of the National Centre for Environmental Prediction of the United States National Weather Service were used (ftp://arlftp.arlhq.noaa.gov/pub/archives/gdas1, accessed on 12 September 2020). Overlay back trajectory maps were compiled for each of the three areas [37] that provided statistical representations of wind movement towards the sampling sites for the aforementioned period.

## 3. Results and Discussion

### 3.1. Cr Source Identification 

#### 3.1.1. Soil

**Figure 3:**
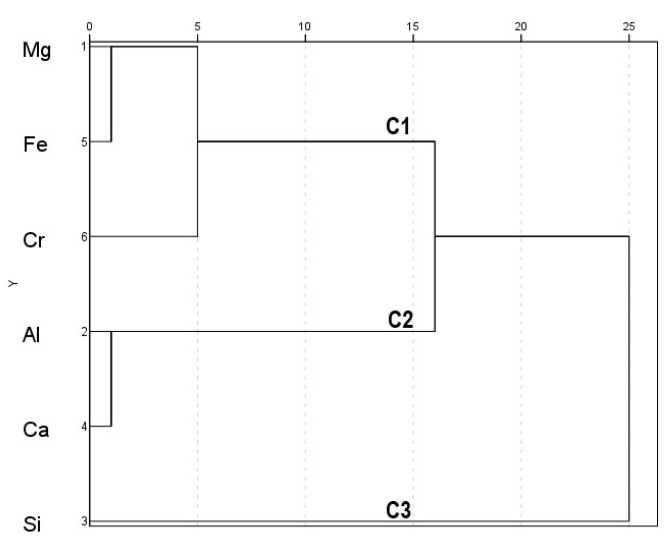
Dendrogram depicting the relationship among the abundant elements detected in the surface soil of the seven sampling sites. C1, Cluster 1; C2, Cluster 2; C3, Cluster 3.

Based on the EDS detected amounts (wt%) of the abundant elements in soil samples (Supplementary Materials, Table S1), correlation coefficient analysis (Table 2a) determined a significant negative correlation between Cr and Ca (r = −0.7406, *p* < 0.05), while Fe had a significant positive correlation with Mg (r = 0.9104, *p* < 0.01) and a negative correlation with Al (r = −0.7945, *p* < 0.01) and Ca (r = −0.9134, *p* < 0.01). In addition, Mg showed a significant negative correlation with Ca (r = −0.7120, *p* < 0.05) and Al (r = −0.9308, *p* < 0.01) (Table 2a). PCA outcomes (Table 3a) showed high positive loadings for Mg and Fe with negative values for Al and Ca on the first principal component (PC1), confirming correlation results. The second principal component (PC2) had negative values for Fe and Cr, but positive loading for Si. PC1 and PC2 represented 46% and 44% of the total variance, respectively, suggesting almost equal contributions from the two components (Table 3a). Cluster analysis formed three clusters for soil elements (Figure 3). Cluster 1 (C1) presented Cr, Fe, and Mg, the second cluster (C2) included Al and Ca, while Si formed the third cluster (C3). This clarified the trends of the PCA outcomes, suggesting a common origin of Cr, Fe, and Mg, and a different source for Al and Ca. Likewise, Si in the surface soil might also be sourced differently from the rest of the elements.

The shared sources of Cr, Fe, and Mg in the surface soils of Sekhukhuneland could be geogenic, i.e., the ultramafic orserpentine substrates that are inherently rich in such elements [38,39]. In this regard, mining-driven overexposure of chromite and other associated minerals of the chromitite seams (which contain high quantities of Cr, Fe and Mg) in the RLS could also have contributed [40]. The possibility of substitution of Fe in chromite by Mg or Ca, and Cr by Fe or Al [41] may further affect concentrations of these elements in the soil. For Al and Ca, another lithological origin could be suggested, but not from chromite, based on the negative association of these two with Cr, Fe, and Mg. Sources of Si could be diverse, including the Si mine near location S8, road dust, and ferrochrome smelter slag [23,29]. Therefore, combined contributions from geogenic and anthropogenic sources could be deduced for the abundant elements in the surface soils of the study region. Disentangling elemental sources in soils could be challenging in urbanized areas such as Sekhukhuneland due to the aggregation of various pollution sources [7,32,42] and high geogenic concentrations of elements [39]. 

#### 3.1.2. Leaf Surfaces

**Figure 4:**
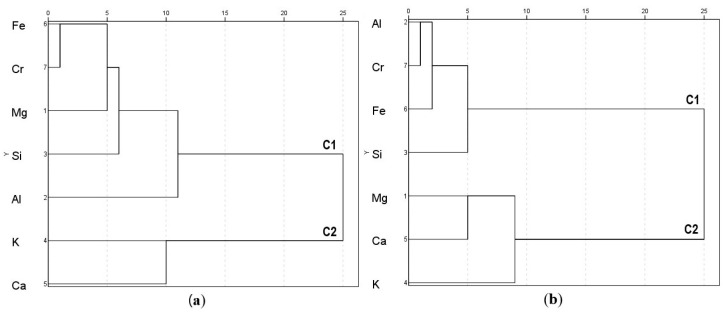
Dendrograms of abundant elements detected on leaf surfaces: (**a**) Adaxial; (**b**) Abaxial.

A summary of amounts (wt%) of the abundant elements detected on the foliar surfaces of the assessed plant species is presented in Table S2, Supplementary Materials. For the adaxial leaf surface, significant positive correlations were determined between Cr and Fe (r = 0.9847, *p* < 0.001), and both of these elements with Al (Cr, r = 0.7881, *p* < 0.01; Fe, r = 0.8573, *p* < 0.01), Mg (Cr, r = 0.8011, *p* < 0.05; Fe, r = 0.8139, *p* < 0.05), and Si (Cr, r = 0.9867, *p* < 0.001; Fe, r = 0.9939, *p* < 0.001) (Table 2b). The PCA substantiated correlations between the same five elements (i.e., Cr, Fe, Mg, Al, and Si) that had high loadings on PC1 and represented 65% of the variance (Table 3b). PC2 had greater loadings for Ca and K, contributing 25% of the total variance. Dendrograms further corroborated PCA outcomes by clustering Cr, Fe, Mg, Al, and Si (C1) separately from Ca and K (C2) (Figure 4a). Correlation, PCA, and cluster analysis results suggested common origins of Cr, Fe, Mg, Al, and Si, whereas Ca and K most likely had other origins. For the abaxial surface, correlation analysis detected a significant positive relation between Cr and Fe (r = 0.8527, *p* < 0.01), Cr and Si (r = 0.8535, *p* < 0.01), and Al and Si (r = 0.8486, *p* < 0.01) (Table 2b). PCA outcomes showed higher loadings for Cr, Fe, Al, and Si on PC1, which contributed 51% of the total variance (Table 3b). Comparatively higher loadings were observed for Mg, Ca, and K under PC2, which represented 32% of the total variance. Cluster analysis was in accord with PCA outcomes showing association among Cr, Fe, Al, and Si that were grouped under C1 (Figure 4b). The remainder of the elements, i.e., Mg, Ca, and K, were included in C2. This means Cr, Fe, Al, and Si could be originated from similar sources, whereas Ca, K, and Mg could have been sourced differently. Non-significant correlations between common elements (i.e., Al, Ca, Cr, Fe, Mg, and Si) detected in soil and the two leaf surfaces (Supplementary Materials, Table S3) could mean negligible contribution from soil to leaf surface elements.

In general, for leaf surfaces, Cr, Fe, Al, Mg, and Si could have been sourced from chromite, which is characterized by the unit formula [(Mg,Fe^2+^)(Al,Cr,Fe^3+^)_2_O_4_] [43]. Moreover, various silicate minerals could be present as dominant gangue minerals in chromite [23,40]. In which case, chromite rich particles dispersed from tailing dams, mining and smelting operations, and ore spillage during transportation [23,44,45] could be the primary dust sources. For the abaxial leaf side, a different origin of Mg compared to the adaxial leaf surface could be related to Mg-richness of the chromitite seams in the RLS and specifically the natural abundance of Mg in the ultramafic soils of Sekhukhuneland [38,39,40]. On the other hand, Ca and K were sourced from other unidentified geogenic (lithogenic) materials. The lack of associations between common elements in soil and leaf surfaces further confirmed a minimum contribution of elements from soil to leaf surface and hence greater possible contribution from atmospheric dust [46]. This also indicated the possible presence of the extremely hazardous Cr(VI) in Cr mine- and ferrochrome smelter-sourced dust particles deposited on plant leaves in Sekhukhuneland [29,30]. Similar to this study, in severely air polluted areas, higher proportions of elements of concern were detected in aerial dust particles compared to the surface soil [47]. For the present study, the found heterogeneity in the origin of the abundant elements on leaf surfaces reflected foliar accumulation of particles with different elemental compositions, which were sourced from varied emitters over time [48].

### 3.2. Leaf Surface–Cr Dust Particle Interactions 

#### 3.2.1. Pearson Correlation Coefficient Analysis 

The highest Cr amounts (Cr wt%) in dust particles and largest Cr particles (CrD) detected on leaf surfaces is presented in Table S4, Supplementary Materials. Among the evaluated plant variables (Supplementary Materials, Table S5), for the adaxial surface, a significant positive correlation was determined between leaf area and Cr wt% (*r* = 0.9870, *p* < 0.001) and CrD (*r* = 0.8692, *p* < 0.001) (Table 4). For the abaxial leaf surface, only CrD was significantly positively correlated with leaf area (*r* = 0.6837, *p* < 0.05) and plant height (*r* = 0.6937, *p* < 0.05). This indicated that both surfaces of larger leaves could be more susceptible to Cr dust deposition, while the abaxial leaf surfaces of taller plants could also be prone to deposition of larger Cr dust particles (Table 4). No significant relations were observed between the pollution variables (i.e., mine or road frequency, Supplementary Materials, Table S6) and Cr wt% and CrD (Table 4). 

#### 3.2.2. Factor Analysis 

For factor analysis (FA), variables with loadings greater than 0.45 were considered in this study. For the adaxial leaf surface, the same group of variables had the highest loadings for Cr wt% and CrD (Table 5). The first factor (F1) indicated high positive loadings for leaf area, epicuticular wax, and plant height, and high negative loadings for stomata density. This suggested that larger leaf area, dense wax structures, plant tallness, and fewer stomata enhanced Cr particle contamination. In the second factor, F2, plant height, and trichome features (size and density) had the highest but negative loadings, of which trichome size had loading > 0.9 for both Cr wt% and CrD. Trichome size was, hence, identified as the pure measure of F2. This indicated a limiting effect of longer trichomes on Cr dust contamination (Table 5). Both factors (F1 and F2) determined a greater association between plant morphological traits and Cr wt% and CrD for the adaxial leaf surface.

Different sets of variables were extracted by FA for the abaxial Cr wt% and CrD (Table 5). Regarding Cr wt%, F1 had the highest positive loadings for stomata size, road frequency, and epicuticular wax, suggesting such variables may favor Cr dust deposition. A negative loading for trichome density means this trait limits abaxial Cr amounts. F2 showed higher but negative loading for trichome size, which indicated a limiting effect of longer trichomes on adhesion of Cr particles. Positive loadings for epicuticular wax and mine frequency under F2 suggested that these variables enhanced Cr particle deposition. Regarding CrD, F1 extracted epicuticular wax, leaf area, stomata size, and mine frequency as variables with higher loadings. Such variables, hence, increase deposition possibility of larger Cr particles. Under F2, negative loadings were observed for stomata size and epicuticular wax. Hence, larger stomata and dense wax structures restricted deposition of larger Cr dust. On the other hand, positive loading for plant height means plant tallness favored adhesion of larger Cr particles. For the abaxial surface, therefore, both pollution and plant variables were associated with Cr wt% and CrD. Some variables, i.e., epicuticular wax, plant height, and stomata size appeared on both factors, indicating a ‘factorially complex’ situation. This indicated that such variables could be moderately predictable under both factors. For the present study, based on the highest loadings of Cr wt% and CrD in F1, the above-mentioned variables were considered only for F1.

In the present study, larger leaf area enhanced Cr dust deposition on both leaf surfaces by presenting greater available surfaces for dust adhesion [49,50,51]. In addition, wax structures on large leaves generally hold dust better compared to smooth leaf surfaces [49], as could also be stated for the plant species investigated in this study. Although longer trichomes restricted Cr dust contamination on both leaf surfaces, denser trichomes increased adhesion possibility of larger Cr particles on the abaxial leaf surfaces. In this case, pubescence could have prevented re-emission of dust particles from the abaxial leaf surfaces [50]. For a chromite mining region in India, presence of trichomes on leaves was identified as the trait to favor dust deposition the most [17]. Similar to this Sekhukhuneland study, both dust adhesion-favoring and limiting effects of trichomes have been suggested in literature [19,46,52,53].

Densely distributed stomata on the adaxial surface further restricted Cr dust contamination in the present study. Hence, it can be assumed that the stomata density did not create the desired adaxial leaf surface roughness that could maximize the trapping of various Cr bearing PM size fractions [22]. However, higher stomata density and larger stomata on the adaxial and abaxial surface, respectively, enhanced deposition of smaller Cr particles. In the past, such features have been reported to create moisture enriched foliar surfaces that favor deposition of finer particles [54].

Plant height was identified as another morphological feature that increased Cr dust particle deposition possibility on both leaf surfaces. This could be explained by the fact that plant tallness creates air turbulence that maximizes aerial PM exposure, resulting in greater deposition on leaves [53,55,56]. This holds for Sekhukhuneland, considering the high density of Cr particles in the regional air mass [23,26].

Besides plant morphological traits, Cr dust deposition on the abaxial leaf surface was further enhanced by the presence of multiple Cr and Pt mines and roads in the vicinity. This suggests that proximity to multiple polluters may increase dust exposure to abaxial leaf surface, which is otherwise less exposed compared to the adaxial surface due to its position, facing down. The higher deposition possibility of re-suspended road dust, especially chromite ore particles dispersed during transportation [23], might further explain the findings of this study. Comparable to this study, previous investigations have also linked greater dust deposition on leaves and other aerial plant parts to proximity to different types of Cr sources such as chromite mines, Cr metal processing industries, quarries, and roads [17,44].

As anticipated for this study, individualistic interaction patterns between plant morphology and pollution sources were recognized for the two leaf surfaces. For the adaxial leaf surface, ‘plant morphology’ and specifically larger leaf area, dense epicuticular wax structures, and plant tallness (plant height) increased Cr particle deposition possibility. In contrast, for the abaxial surface, leaf morphology (i.e., larger leaf area, larger stomata, and dense epicuticular wax structures) and pollution sources (i.e., proximity to and number of mines and roads) conjointly maintained the ‘leaf surface–dust interface’, which predominantly controlled Cr dust deposition. 

For Sekhukhuneland, presence of various microstructures on larger leaves could be the key factor that increased Cr dust adhesion possibility [1]. This is because various micromorphological features add structural heterogeneity (surface roughness) to foliar contact surfaces, which favored the holding of Cr particles of different size fractions [15,22]. Various plant morphological traits, therefore, cohesively affected foliar Cr dust deposition rather than individual dominance under the influence of ambient air pollution [15,17,20,21,50,51]. However, because EDS analysis presents semi-quantitative data, future studies should select other quantitative approaches to determine the elemental composition of road, atmospheric, and leaf surface dust particles. In addition, a wider variety of plant species should be assessed to make more informed predictions in this regard. Nevertheless, leaves acted as functional interfaces between plants and polluted urban environments in the study region [1,57] and showed dust mitigation prospects. As each leaf surface interacts differently with the pollution factor, dust capture capacity of both leaf surfaces should be considered to estimate the PM mitigation potential of plant species. 

### 3.3. Air Mass Movement Patterns 

**Figure 5:**
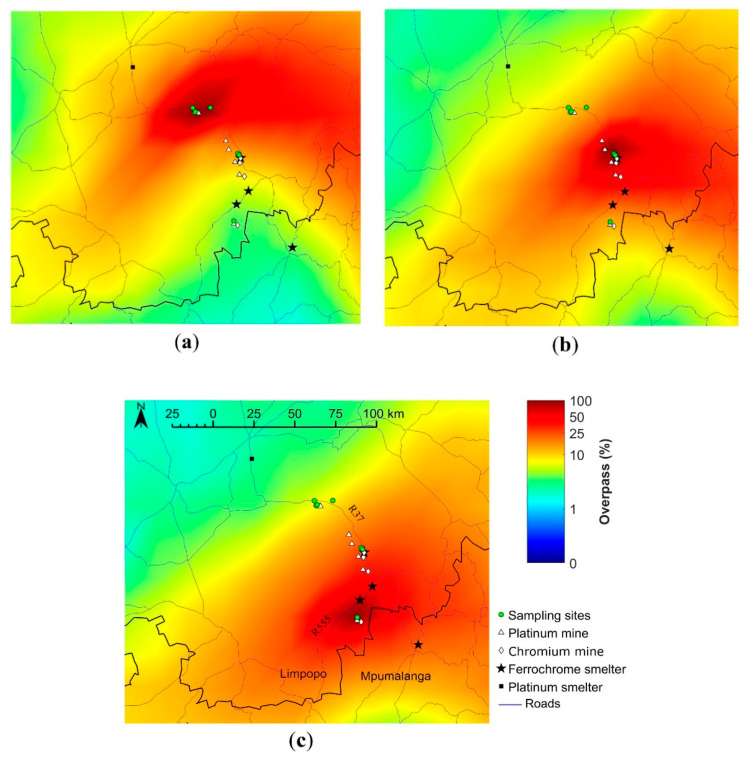
Overlay back trajectory maps calculated over the period 1 April 2018 to 30 November 2018 for the study region sectioned in: (**a**) Area 1 (incorporated sampling sites S3–S5, S7); (**b**) Area 2 (S1, S2); (**c**) Area 3 (S8). The color code represents the percentage of the trajectories passing over 0.2° × 0.2° grid cells superimposed on the regional map. The colors red and blue indicate the highest and lowest percentage overpasses, respectively.

As is evident from the air mass history for Area 1, dust generated in relative proximity (around 20–25 km, indicated by the darker red zone in Figure 5a) in all directions of the sampling areas, could have contributed the most to deposited dust on foliar surfaces. One of the main roads (R37) and a Pt mine lie within this specific air mass fetch region and therefore could be identified as likely dust polluters in Area 1. Dust generated in a larger zone between northeast and southeast, as well as a smaller zone between southwest and west (both indicated by the less intense shades of red in Figure 5a) might have contributed as well. However, no Cr polluters were located in this fetch region, therefore, the dust could be of unknown origin. Sources occurring in the fetch region with lower air mass overpass frequencies (indicated with yellow and green color in Figure 5a), especially the Pt smelter in Polokwane, are likely to contribute less towards dust pollution in Area 1.

The air mass histories for Areas 2 and 3 (Figure 5b,c) indicated similar patterns to that observed for Area 1 (Figure 5a), with maximum dust contributions from local polluters located within the 20–25 km periphery (dark red zones in the maps) with some regional contributions as indicated by the large fetch region (less intense red shaded zone in Figure 5b,c). For Area 2, multiple mines (four Pt and two Cr mines), one active ferrochrome smelter, and the main road R37 occur in the air mass fetch region with the highest overpass. Similarly, one of each Pt and Cr mine, a ferrochrome smelter along with another main road (R555) are located within the fetch region with the highest overpass for Area 3. The other ferrochrome smelter located in the larger fetch region (less intense shades of red in Figure 5c) is likely to make a less significant dust contribution to Area 3.

Air mass movement patterns indicated the dominance of local Cr sources as dust polluters in this mining–smelting industrial region of Sekhukhuneland. Wind movement patterns, hence, further substantiated the source identification and leaf surface–Cr dust particle assessment outcomes of this study. Therefore, wind movements showed considerable influence on the spatial and temporal dust dispersion patterns in Sekhukhuneland [23,26]. Furthermore, from the back trajectory maps, it can be deduced that Cr dust pollution is greatest within the 20 to 25 km periphery around the major emitters, which indicates a need to implement dust mitigation plans.

As is the case with most air polluted urban spaces, creating green barriers and green walls between the polluters and settlements and planting trees along the main roads could present feasible solutions for Sekhukhuneland [15,22,46,51,58]. However, it is essential to identify air pollution-tolerant indigenous plant species with different dust deposition and retention-favoring morphological traits to achieve mitigation at a greater level [16,18,22,51]. By improving the air quality, green spaces may also reduce human exposure to hazardous dust particles [6,22], thereby supporting sustainable urban development near industrial regions of Sekhukhuneland.

## 4. Conclusions

The study established that anthropogenic sources predominantly determine Cr dust deposition on leaf surfaces in the mining–smelting industrial region of Sekhukhuneland. Association between Cr, Fe, Mg, Al, and Si further identified chromite containing dust particles, probably generated by mining–smelting–transportation activities and from tailings, as the primary sources of these elements on leaf surfaces. Moreover, lack of significant relations between the common elements (Al, Ca, Cr, Fe, Mg, and Si) in soil and leaf surfaces confirmed negligible contribution from soil to leaf surface elements. Ultramafic outcrops and mining related overexposure of chromite and other Cr rich minerals present in the chromitite seams of the RLS could be the most likely sources of Cr in the surface soils. The two leaf surfaces interacted differently with dust pollution. For the adaxial surface, plant morphological traits such as plant height, larger leaf area, and dense epicuticular wax structures predominantly increased Cr dust contamination possibility. Proximity to several roads and mines and leaf macro- and micromorphology, i.e., larger leaf area, dense epicuticular wax structures, and larger stomata conjointly enhanced Cr particle deposition on the abaxial leaf surface. Hence, larger leaves with various micromorphologies could present the most favorable interfaces for Cr dust particle deposition. Air mass movement patterns further confirmed local Cr sources in Sekhukhuneland, i.e., mines, ferrochrome smelters, and main roads as the major dust generators causing severe pollution within 20 to 25 km periphery. Both plant morphology and pollution factors, therefore, need careful consideration to comprehend foliar dust contamination in air polluted localities. Common plants with dust adhesion-favoring morphological traits seem to be ideal PM mitigation agents to improve air quality and lower human health risks in populated urban environments associated with mining and smelting localities of South Africa.

## Figures and Tables

**Table 1:** Sampling sites and location of Cr and Pt mines within 20 km and roads within 110 m radius.

Sampling Site	Plant Species	Cr Sources	
		Mines (km)	Roads (m)
^††^ S1	*Argemone ochroleuca* *Gomphocarpus fruticosus*	4.1, 7.4 (Cr); 10.1 (Pt)	24, 29.4
^†††^ S2	*Carica papaya* *Catharanthus roseus* *Psidium guajava* *Senna italica*	2.8 (Cr); 9.8, 12.4, 16 (Pt)	13.6
S3	*Citrus limon* *Ipomoea batatas*	17.9 (Pt)	104.1
S4	*Peltophorum africanum*	3.1 (Pt)	4.7, 44.2
S5	*Tribulus terrestris*	7 (Pt)	4.1, 20.5
S7	*Moringa oleifera*	2.4 (Pt)	4.9, 23.1
^†^ S8	*Ozoroa paniculosa*	4.1 (Cr); 3.8 (Pt)	64.2

Sites close to ferrochrome smelters are indicated as follows: ^†††^ < 3 km; ^††^ 3–5 km and ^†^ 5–13 km.

**Table 2:** Pearson correlation coefficient matrices for detected elements (**a**) in surface soil and (**b**) on leaf surfaces.

a.	Cr	Fe	Mg	Al	Si	Ca
Fe	0.641					
Mg	0.5429	0.9104 **				
Al	−0.5888	−0.7945 *	−0.9308 **			
Si	−0.6768	0.0594	0.1617	0.0738		
Ca	−0.7406*	−0.9134 **	−0.7120*	0.6783	0.2486	
**b.**						
Fe	0.9847 ^ad^*** 0.8527 ^ab^**					
Mg	0.8011 ^ad^* −0.0328 ^ab^	0.8139 ^ad^* 0.0198 ^ab^				
Al	0.7881 ^ad^** 0.8012 ^ab^*	0.8573 ^ad^** 0.6864 ^ab^	0.6785 ^ad^ −0.0260 ^ab^			
Si	0.9867 ^ad^*** 0.8535 ^ab^**	0.9939 ^ad^*** 0.7225 ^ab^*	0.8405 ^ad^** 0.1112 ^ab^	0.8677 ^ad^** 0.8486 ^ab^**		
Ca	0.1699 ^ad^ 0.0334 ^ab^	−0.0923 ^ad^ −0.2952 ^ab^	0.3205 ^ad^ 0.6999 ^ab^	0.1057 ^ad^ 0.0794 ^ab^	−0.07260 ^ad^ 0.1478 ^ab^	
K	0.4202 ^ad^ −0.3533 ^ab^	0.4755 ^ad^ −0.4097 ^ab^	0.4803 ^ad^ 0.6010 ^ab^	0.4356 ^ad^ −0.6647 ^ab^	0.4420 ^ad^ −0.2976 ^ab^	0.5990 ^ad^ 0.5254 ^ab^

Superscripts: ad, adaxial; ab, abaxial values. Significance, * = *p* < 0.05; ** = *p* < 0.01; *** = *p* < 0.001. K was not detected in soils from all sampling sites, hence excluded from this analysis.

**Table 3:** Varimax rotated PCA results of abundant elements detected (**a**) in surface soil and (**b**) on leaf surfaces. Loadings higher than 0.5 are considered.

Elements	a. Soil		b. Leaf Surfaces			
			Ad		Ab	
	PC1	PC2	PC1	PC2	PC1	PC2
Al	−0.938	0.103	0.866	0.180	0.932	−0.096
Ca	−0.690	0.589	−0.128	0.963	0.052	0.895
Cr	0.145	−0.962	0.987	−0.023	0.940	−0.017
Fe	0.620	−0.723	0.994	0.054	0.860	−0.172
K	-	-	0.404	0.789	−0.471	0.754
Mg	0.968	0.201	0.816	0.376	0.066	0.903
Si	0.267	0.901	0.998	0.055	0.933	0.122
Eigenvalue	2.768	2.657	4.554	1.729	3.591	2.239
Cumulative %	46.130	90.416	65.057	89.764	51.294	83.275

Ad, adaxial; Ab, abaxial; PC1, principal component 1; PC 2, principal component 2. K was not detected in soils from all sampling sites, hence excluded from this analysis.

**Table 4:** Pearson correlation coefficient matrices of selected variables (plant morphology and pollution sources) with the highest Cr amounts in dust particles (Cr wt%) and largest Cr dust particles (CrD) detected on adaxial (Ad) and abaxial (Ab) leaf surfaces.

Variables	Ad		Ab	
	Cr wt%	CrD	Cr wt%	CrD
Mine frequency	0.2987	0.3590	0.2291	0.3195
Road frequency	−0.431	0.1036	0.5459	0.1013
Plant height	0.3103	0.3646	0.2271	0.6937 *
Leaf area	0.9870 ***	0.8692 ***	−0.2652	0.6837 *
Epicuticular wax	0.3903	0.4084	0.5404	0.4184
Stomata size	−0.2404	−0.4042	0.5373	−0.1471
Stomata density	−0.2808	−0.4634	0.1008	0.4075
Trichome size	−0.2971	−0.1172	0.0206	−0.1929
Trichome density	−0.3155	0.0595	−0.2390	0.1736

Significance, * = *p* < 0.05; *** = *p* < 0.001.

**Table 5:** Varimax rotated FA matrix for, (**a**) highest Cr amount in dust particles (Cr wt%) and (**b**) largest Cr dust particles (CrD).

a.	Ad		Ab		b.	Ad		Ab	
	Factor 1	Factor 2	Factor 1	Factor 2		Factor 1	Factor 2	Factor 1	Factor 2
Cr wt%	0.874	0.215	0.652	0.071	CrD	0.890	0.004	0.915	0.367
Mine frequency	0.298	0.028	0.256	0.458	Mine frequency	0.333	0.056	0.461	−0.184
Road frequency	−0.172	0.196	0.705	−0.372	Road frequency	−0.102	−0.235	0.055	−0.311
Plant height	0.485	−0.696	−0.243	0.120	Plant height	0.477	−0.607	0.433	0.687
Leaf area	0.848	0.208	−0.098	0.342	Leaf area	0.751	0.231	0.571	0.136
Epicuticular wax	0.515	0.196	0.659	0.712	Epicuticular wax	0.556	0.290	0.757	−0.507
Stomata size	−0.382	0.300	0.738	0.171	Stomata size	−0.429	0.253	0.450	−0.734
Stomata density	−0.584	0.238	−0.003	0.251	Stomata density	−0.666	0.131	0.277	−0.034
Trichome size	−0.190	−0.916	0.109	−0.839	Trichome size	−0.127	−0.996	−0.389	0.084
Trichome density	−0.135	−0.598	−0.598	−0.036	Trichome density	−0.021	−0.575	−0.056	0.802
Eigenvalue	2.645	1.996	2.404	1.753	Eigenvalue	2.664	1.972	2.400	2.202
Cumulative %	26.449	46.405	24.040	41.571	Cumulative %	26.641	46.367	24.006	46.027

Ad, adaxial; Ab, abaxial.Loadings > 0.45 are considered.

## Data Availability

Not applicable.

## Supplementary Materials

The supporting information can be downloaded at: https://www.mdpi.com/article/10.3390/ijerph19042072/s1. Table S1: EDS analysis detected abundant elements (wt%) in the soils of the seven sampling sites; Table S2: EDS analysis detected abundant elements (wt%) on plant leaf surfaces; Table S3: Pearson correlation matrix of major elements detected in soil and (a) adaxial and (b) abaxial leaf surface; Table S4: EDS detected highest Cr amounts in dust particles (Cr wt%) and largest Cr particles (CrD) identified on each of the leaf surfaces per species; Table S5: Evaluated plant morphological features; Table S6: Mine and road frequency index.

## References

[1] Zhang W., Wang B., Niu X. (2017). Relationship between leaf surface characteristics and particle capturing capacities of different tree species in Beijing. Forests.

[2] Franchini M., Mannucci P.M. (2018). Mitigation of air pollution by greenness: A narrative review. Eur. J. Intern. Med..

[3] Entwistle J.A., Hursthouse A.S., Marinho Reis P.A., Stewart A.G. (2019). Metalliferous mine dust: Human health impacts and the potential determinants of disease in mining communities. Curr. Pollut. Rep..

[4] Balabanova B., Stafilov T., Šajn R., Bačeva K. (2012). Distribution of chemical elements in attic dust as reflection of their geogenic and anthropogenic sources in the vicinity of the copper mine and flotation plant. Arch. Environ. Contam. Toxicol..

[5] Csavina J., Field J., Taylor M.P., Gao S., Landázuri A., Betterton E.A., Eduardo Sáez A. (2012). A review on the importance of metals and metalloids in atmospheric dust and aerosol from mining operations. Sci. Total Environ..

[6] Gil-Loaiza J., Field J.P., White S.A., Csavina J., Felix O., Betterton E.A., Eduardo Sáez A., Maier R.M. (2018). Phytoremediation reduces dust emissions from metal(loid)-contaminated mine tailings. Environ. Sci. Technol..

[7] Gabarrón M., Faz A., Acosta J.A. (2018). Use of multivariable and redundancy analysis to assess the behavior of metals and arsenic in urban soil and road dust affected by metallic mining as a base for risk assessment. J. Environ. Manag..

[8] Ebenebe P.C., Shale K., Sedibe M., Tikilili P., Achilonu M.C. (2017). South African mine effluents: Heavy metal pollution and impact on the ecosystem. Int. J. Chem. Sci..

[9] Tian S., Liang T., Li K. (2019). Fine road dust contamination in a mining area presents a likely air pollution hotspot and threat to human health. Environ. Int..

[10] Mhlongo S.E., Amponsah-Dacosta F. (2015). A review of problems and solutions of abandoned mines in South Africa. Int. J. Min. Reclam. Environ..

[11] Amoah P., Eweje G. (2021). Impact mitigation or ecological restoration? Examining the environmental sustainability practices of multinational mining companies. Bus. Strat. Environ..

[12] WHO (2016). Ambient Air Pollution: A Global Assessment of Exposure and Burden of Disease. https://apps.who.int/iris/bitstream/handle/10665/250141/9789241511/9789241511/9789241511353-eng.pdf?sequence=1.

[13] Das A., Kumar R., Patel S.S., Saha M.C., Guha D. (2020). Source apportionment of potentially toxic elements in street dust of a coal mining area in Chhattisgarh, India, using multivariate and lead isotopic ratio analysis. Environ. Monit. Assess..

[14] Song Y., Huang B., He Q., Chen B., Wei J., Mahmood R. (2019). Dynamic assessment of PM_2.5_ exposure and health risk using remote sensing and geo-spatial big data. Environ. Pollut..

[15] Ysebaert T., Koch K., Samson R., Denys S. (2021). Green walls for mitigating urban particulate matter pollution—A review. Urban For. Urban Green..

[16] Javanmard Z., Kouchaksaraei M.T., Hosseini S.M., Pandey A.K. (2020). Assessment of anticipated performance index of some deciduous plant species under dust air pollution. Environ. Sci. Pollut. Res..

[17] Mandal K., Dhal N.K. (2021). Pollution resistance assessment of plants around chromite mine based on anticipated performance index, dust capturing capacity and metal accumulation index. Res. Sq..

[18] Mondal S., Singh G. (2021). Air pollution tolerance, anticipated performance, and metal accumulation capacity of common plant species for green belt development. Environ. Sci. Pollut. Res..

[19] Perini K., Ottelé M., Giulini S., Magliocco A., Roccotiello E. (2017). Quantification of fine dust deposition on different plant species in a vertical greening system. Ecol. Eng..

[20] Weerakkody U., Dover J.W., Mitchell P., Reiling K. (2018). Evaluating the impact of individual leaf traits on atmospheric particulate matter accumulation using natural and synthetic leaves. Urban For. Urban Green..

[21] Chaudhary I.J., Rathore D. (2019). Dust pollution: Its removal and effect on foliage physiology of urban trees. Sustain. Cities Soc..

[22] Redondo-Bermúdez M.C., Gulenc I.T., Cameron R.W., Inkson B.J. (2021). Green barriers’ for air pollutant capture: Leaf micromorphology as a mechanism to explain plants capacity to capture particulate matter. Environ. Pollut..

[23] Tshehla C., Djolov G. (2018). Source profiling, source apportionment and cluster transport analysis to identify the sources of PM and the origin of air masses to an industrialised rural area in Limpopo. Clean Air J..

[24] Nkosi V., Wichmann J., Voyi K. (2015). Mine dumps, wheeze, asthma, and rhinoconjunctivitis among adolescents in South Africa: Any association?. Int. J. Environ. Health Res..

[25] Nkosi V., Wichmann J., Voyi K. (2017). Indoor and outdoor PM_10_ levels at schools located near mine dumps in Gauteng and North West Provinces, South Africa. BMC Public Health.

[26] Tshehla C., Wright C.Y. (2019). Spatial variability of PM_10_, PM_2.5_ and PM chemical components in an industrialised rural area within a mountainous terrain. S. Afr. J. Sci..

[27] Scholes R.J., Biggs R. (2005). A biodiversity intactness index. Nature.

[28] Adhikari S., Siebert S.J., Jordaan A. (2021). Evidence of chromium dust pollution on the leaves of food and medicinal plants from mining areas of Sekhukhuneland, South Africa. S. Afr. J. Bot..

[29] Coetzee J.J., Bansal N., Chirwa E.M.N. (2018). Chromium in environment, its toxic effect from chromite-mining and ferrochrome industries, and its possible bioremediation. Expos. Health.

[30] Das P.K., Das B.P., Dash P. (2021). Chromite mining pollution, environmental impact, toxicity and phytoremediation: A review. Environ. Chem. Lett..

[31] Cramer L.A., Basson J., Nelson L.R. (2004). The impact of platinum production from UG2 ore on ferrochrome production in South Africa. J. South. Afr. Inst. Min. Metall..

[32] Shi G., Chen Z., Xu S., Zhang J., Wang L., Bi C., Teng J. (2008). Potentially toxic metal contamination of urban soils and roadside dust in Shanghai, China. Environ. Pollut..

[33] Norouzi S., Khademi H. (2015). Source identification of heavy metals in atmospheric dust using *Platanus orientalis* L. leaves as bioindicator. Eurasian J. Soil Sci..

[34] Du Toit M.J., Cilliers S.S. (2010). Aspects influencing the selection of representative urbanization measures to quantify urban-rural gradients. Landsc. Ecol..

[35] Liu L., Guan D., Peart M.R. (2012). The morphological structure of leaves and the dust-retaining capability of afforested plants in urban Guangzhou, South China. Environ. Sci. Pollut. Res. Int..

[36] Draxler R.R., Hess G.D. (2004). Description of the HYSPLIT 4 Modelling System. National Oceanic and Atmospheric Administration (NOAA) Technical Memorandum ERL ARL–224. https://www.arl.noaa.gov/data/web/models/hysplit4/win95/arl-224.pdf.

[37] Vakkari V., Laakso H., Kulmala M., Laaksonen A., Mabaso D., Molefe M., Kgabi N., Laakso L. (2011). New particle formation events in semi-clean South African savannah. Atmos. Chem. Phys..

[38] Siebert S.J., Van Wyk A.E., Bredenkamp G.J. (2002). The physical environment and major vegetation types of Sekhukhuneland, South Africa. S. Afr. J. Bot..

[39] Adhikari S., Marcelo-Silva J., Rajakaruna N., Siebert S.J. (2022). Influence of land use and topography on distribution and bioaccumulation of potentially toxic metals in soil and plant leaves: A case study from Sekhukhuneland, South Africa. Sci. Total Environ..

[40] Naldrett A.J., Wilson A., Kinnaird J., Yudovskaya M., Chunnett G. (2012). The origin of chromitites and related PGE mineralization in the Bushveld Complex: New mineralogical and petrological constraints. Miner. Depos..

[41] Gu F., Wills B.A. (1988). Chromite—Mineralogy and processing. Miner. Eng..

[42] Oliva S.R., Espinosa F.A.F. (2007). Monitoring of heavy metals in topsoils, atmospheric particles and plant leaves to identify possible contamination sources. Microchem. J..

[43] Haggerty S.E. (1991). Oxide mineralogy of the upper mantle. Rev. Mineral. Geochem..

[44] Pöykiö R., Mäenpä A., Perämäki P., Niemelä M., Välimäki I. (2005). Heavy metals (Cr, Zn, Ni, V, Pb, Cd) in Lingonberries (*Vaccinium vitis-idaea* L.) and assessment of human exposure in two industrial areas in the Kemi-Tornio region, northern Finland. Arch. Environ. Contam. Toxicol..

[45] Özgen S. (2012). Modelling and optimization of clean chromite production from fine chromite tailings by a combination of multi gravity separator and hydro cyclone. J. South. Afr. Inst. Min. Metall..

[46] Gajbhiye T., Pandey S.K., Kim K.H., Szulejko J.E., Prasad S. (2016). Airborne foliar transfer of PM bound heavy metals in *Cassia siamea*: A less common route of heavy metal accumulation. Sci. Total Environ..

[47] Tang Y., Han G. (2015). Characteristics of major elements and heavy metals in atmospheric dust in Beijing, China. J. Geochem. Explor..

[48] Yang J., Teng Y., Song L., Zuo R. (2016). Tracing sources and contamination assessments of heavy metals in road and foliar dusts in a typical mining city, China. PLoS ONE.

[49] Nguyen T., Yu X., Zhang Z., Liu M., Liu X. (2015). Relationship between types of urban forest and PM_2.5_ capture at three growth stages of leaves. J. Environ. Sci..

[50] Leonard R.J., McArthur C., Hochuli D.F. (2016). Particulate matter deposition on roadside plants and the importance of leaf trait combinations. Urban For. Urban Green..

[51] Roy A., Bhattacharya T., Kumari M. (2020). Air pollution tolerance, metal accumulation and dust capturing capacity of common tropical trees in commercial and industrial sites. Sci. Total Environ..

[52] El-Khatib A.A., Abdel-Rahman A.M., El-Sheikh O.M. (2011). Leaf geometric design of urban trees: Potentiality to capture airborne particle pollutants. J. Environ. Stud..

[53] Ram S.S., Majumder S., Chaudhuri P., Chanda S., Santra S.C., Maiti P.K., Sudarshan M., Chakraborty A. (2012). Plant canopies: Bio-monitor and trap for re-suspended dust particulates contaminated with heavy metals. Mitig. Adapt. Strateg. Glob. Chang..

[54] Zha Y., Shi Y., Tang J., Liu X., Feng C., Zhang Y. (2019). Spatial-temporal variability and dust-capture capability of 8 plants in urban China. Pol. J. Environ. Stud..

[55] Tallis M., Taylor G., Sinnett D., Freer-Smith P. (2011). Estimating the removal of atmospheric particulate pollution by the urban tree canopy of London, under current and future environments. Landsc. Urban Plan..

[56] Mo L., Ma Z., Xu Y., Sun F., Lun X., Liu X., Chen J., Yu X. (2015). Assessing the capacity of plant species to accumulate particulate matter in Beijing, China. PLoS ONE.

[57] Neinhuis C., Barthlott W. (1998). Seasonal changes of leaf surface contamination in beech, oak, and ginkgo in relation to leaf micromorphology and wettability. New Phytol..

[58] Wang H., Shi H. (2021). Particle retention capacity, efficiency, and mechanism of selected plant species: Implications for urban planting for improving urban air quality. Plants.

