# Supplementary material for: Anthropogenic Sources Dominate Foliar Chromium Dust Deposition in a Mining-Based Urban Region of South Africa
Source: Int J Environ Res Public Health. 2022 Feb 12;19(4):2072. doi: 10.3390/ijerph19042072 (PMC8872262; doi:10.3390/ijerph19042072)
Supplement: Supplementary file 1 [file ijerph-19-02072-s001.zip › ijerph-1539609-supplementary.pdf]

Anthropogenic sources dominate foliar chromium dust deposition in a mining based urban region of South Africa

Sutapa Adhikari [1,*], Anine Jordaan [2], Johan Paul Beukes [3], Stefan John Siebert [1]

Supplementary Materials

Table S1. EDS analysis detected abundant elements (wt%) in the soils of the seven sampling sites.

Elements	S1	S2	S3	S4	S5	S7	S8
Al	3.05	3.1	1.42	8.46	4.1	5.81	9.56
Ca	1.32	0.95	1.3	7.0	0.74	5.44	2.29
Cr	8.93	4.02	0.4	0.001	18.45	0.001	3.62
Fe	8.12	9.4	8.42	3.96	13.37	3.33	5.53
Mg	8.7	10.1	12.12	4.08	4.93	3.36	1.57
Si	11.89	18.16	20.22	19.37	10.53	15.03	13.77

Table S2. EDS analysis detected abundant elements (wt%) on plant leaf surfaces. Ad, adaxial; Ab, abaxial.

Elements		Cr	Fe	Al	Si	Ca	Mg	K
C. limon	Ad	0.001	0.19	0.10	0.39	0.47	0.29	0.79
	Ab	0.001	0.001	0.001	0.23	0.41	0.30	0.68
C. papaya	Ad	1.56	2.12	0.41	2.82	0.55	3.21	1.82
	Ab	0.001	0.001	0.001	0.31	1.50	1.84	2.11
M. oleifera	Ad	0.001	0.001	0.001	0.21	1.43	4.47	0.80
	Ab	0.16	0.22	0.14	0.38	1.07	3.37	0.81
O. paniculosa	Ad	0.13	0.001	0.001	0.18	0.001	2.71	0.10
	Ab	0.001	0.001	0.001	0.13	0.001	0.001	0.40
P. africanum	Ad	0.11	0.30	0.21	0.56	0.86	0.49	0.14
	Ab	0.28	0.40	0.30	0.80	0.89	2.03	0.25
P. guajava	Ad	0.07	0.19	0.09	0.28	0.10	6.86	0.001
	Ab	0.15	0.30	0.18	0.62	0.28	6.40	0.35
A. ochroleuca	Ad	0.21	0.37	0.26	0.75	0.21	4.68	1.36
	Ab	0.09	0.17	0.13	0.42	0.12	4.01	1.47
G. fruticosus	Ad	0.11	0.35	0.14	0.42	0.41	4.46	2.57
	Ab	0.17	0.27	0.001	0.41	0.59	2.45	1.85
C. roseus	Ad	0.14	0.36	0.17	0.58	0.60	4.09	0.24
	Ab	0.12	0.32	0.12	0.31	0.44	3.98	0.47
I. batatas	Ad	0.001	0.47	0.27	1.58	0.19	1.75	2.03
	Ab	0.001	0.78	0.32	1.98	0.001	1.54	2.30
S. italica	Ad	0.001	0.001	0.001	0.14	0.61	0.67	1.58
	Ab	0.001	0.001	0.001	0.23	0.72	0.68	1.37
T. terrestris	Ad	0.001	0.001	0.08	0.24	2.69	1.51	1.15
	Ab	0.001	0.001	0.001	0.11	3.31	0.88	1.23

Table S3. Pearson correlation matrix of major elements detected in soil and (a) adaxial and (b) abaxial leaf surface. Significant at $p < 0.05$.

**a.**	**Mg**	**Al**	**Si**	**Ca**	**Fe**	**Cr**
Mg	0.3052	-0.3823	0.2014	-0.0193	0.1280	-0.1033
	p=0.462	p=0.350	p=0.633	p=0.964	p=0.763	p=0.808
Al	00.6029	-0.4599	0.2608	-0.2649	0.5017	0.2755
	p=0.114	p=0.252	p=0.533	p=0.526	p=0.205	p=0.509
Si	0.4461	-0.3496	0.3004	-0.2888	0.4232	0.0764
	p=0.268	p=0.396	p=0.470	p=0.488	p=0.296	p=0.857
Ca	0.0214	-0.2767	-0.4849	0.2838	-0.2715	0.2422
	p=0.960	p=0.507	p=0.223	p=0.496	p=0.515	p=0.563
Fe	0.4921	-0.3881	0.3104	-0.3294	0.4715	0.1035
	p=0.215	p=0.342	p=0.454	p=0.426	p=0.238	p=0.807
Cr	0.3790	-0.2838	0.2772	-0.3187	0.4075	0.0547
	p=0.354	p=0.496	p=0.506	p=0.442	p=0.316	p=0.898
**b.**						
Mg	0.3867	-0.4061	0.4430	0.0677	0.1472	-0.2374
	p=0.344	p=0.318	p=0.272	p=0.873	p=0.728	p=0.571
Al	-0.1208	0.2053	0.5143	0.6405	-0.3735	-0.5094
	p=0.776	p=0.626	p=0.192	p=0.087	p=0.362	p=0.197
Si	0.1054	0.0050	0.4508	0.5009	-0.1781	-0.2678
	p=0.804	p=0.991	p=0.262	p=0.206	p=0.673	p=0.521
Ca	0.2482	-0.3533	-0.0418	0.1225	0.0019	0.0892
	p=0.553	p=0.391	p=0.922	p=0.773	p=0.996	p=0.834
Fe	0.0298	0.0550	0.4967	0.4700	-0.2055	-0.3964
	p=0.944	p=0.897	p=0.211	p=0.240	p=0.625	p=0.331
Cr	-0.0719	0.0683	0.1930	0.6221	-0.3853	-0.2384
	p=0.866	p=0.872	p=0.647	p=0.100	p=0.346	p=0.570

Table S4. EDS detected highest Cr amounts in dust particles (Cr wt%) and largest Cr particles (CrD) identified on each of the leaf surfaces per species. Ad, adaxial; Ab, abaxial.

Plant species	Cr wt%		CrD
C. papaya	Ad	13.46	81.16
	Ab	0.21	30.89
M. oleifera	Ad	0.49	25.85
	Ab	23.03	15.37
P. guajava	Ad	0.4	8.64
	Ab	1.78	20.89
O. paniculosa	Ad	0.2	6.02
	Ab	0.75	14.77
C. roseus	Ad	23.17	12.39
	Ab	13.72	1.87
A. ochroleuca	Ad	20.8	6.79
	Ab	18.87	7.53
P. africanum	Ad	5.39	4.29
	Ab	14.9	6.58
G. fruticosus	Ad	1.47	3.8
	Ab	9.52	1.89

Table S5. Evaluated plant morphological features. All measurements are given as mean values except epicuticular wax for which wax structural density scores are tabulated. *Not observed due to wax, ** not detected due to puberulous surface. Ad, adaxial; Ab, abaxial.

Plant Species	Plant height (m)	Leaf area (cm)	Stomata				Trichome				Epicuticular wax structural density	
			Size (length, μm)		Density (mm^{-2})		Size (length, μm)		Density (mm^{-2})			
			Ad	Ab	Ad	Ab	Ad	Ab	Ad	Ab	Ad	Ab
C. limon	0.99	11.31	a	4.63	a	81	a	a	a	a	2	1
C. papaya	6.87	669.68	a	6.61	a	68	a	a	a	a	4	4
M. oleifera	4.93	0.81	a	7.29	a	32	81.51	a	13	a	4	4
O. paniculosa	11.76	4.08	×	××	×	××	218.9	118.96	2.6	484	2	1
P. africanum	8.82	0.20	14.06	12.9	9	26	152.6	316.87	9	33	2	4
P. guajava	4.39	23.90	a	8.05	a	146	171.75	188.41	8	18	2	3
G. fruticosus	1.45	3.47	18.59	14.98	5	6	72.13	101.93	8	4	2	3
A. ochroleuca	0.76	9.17	15.45	22.54	15	15	a	a	a	a	4	4
C. roseus	0.53	4.91	8.95	8.35	14	103	a	a	a	a	3	3
I. batatas	0.19	3.32	13.39	15.06	22	45	a	a	a	a	2	2
S. italica	0.17	1.07	5.39	6.38	16	11	60.24	45.54	7	12	4	4
T. terrestris	0.07	0.10	6.12	5.79	47	13	116.85	708.69	4	12	0	0
Range	0.07–11.76	0.1–669.68	5.39–18.59	4.63–22.54	5–47	6–146	60.24–218.9	45.54–708.69	2.6–13	4–484	-	-

Table S6. Mine and road frequency index. Total scores per site are given in bold.

Distance range	Score	S1	S2	S3	S4	S5	S7	S8
**a. Mine frequency index (km)**								
0—3	5	0	1x5	0			1x5	
3—6	4	1x4	0	0	1x4			2x4
6—9	3	1x3	0	0		1x3		
9—15	2	2x2	2x2	0				
15—20	1	2x1	1x1	1x1				
**Total score**	**15**	**13**	**10**	**1**	**4**	**3**	**5**	**8**
**b. Road frequency index (m)**								
0—5	5				1x5	1x5	1x5	
5—15	4		1x4					
15—25	3	1x3				1x3	1x3	
25—45	2	2x2			1x2			
45—105	1			1x1				1x1
**Total score**	**15**	**5**	**4**	**1**	**7**	**8**	**8**	**1**
